# Protective effects of melatonin on myocardial microvascular endothelial cell injury under hypertensive state by regulating Mst1

**DOI:** 10.1186/s12872-023-03159-1

**Published:** 2023-04-01

**Authors:** Lingpeng Wang, Wei Wang, Ruimei Han, Yang Liu, Bin Wu, Jian Luo

**Affiliations:** 1 Department of Cardiology, the First Affiliated Hospital, Xinjiang Medical University, Urumqi, 830000 China; 2 Department of Internal Medicine, The First Affiliated Hospital, Xinjiang Medical University, No. 137, Liyushan South Road, Urumqi, Xinjiang 830000 China; 3 Department of Cardiology, Shanghai Xuhui Central Hospital, Shanghai, 200031 China; 4 Department of Geriatrics, Xinjiang Military General Hospital, 359 Youhao North Street, Urumqi, Xinjiang 830000 China

**Keywords:** Melatonin, Mammalian STE20-like kinase 1 (Mst1), Hypertension, Myocardial injury, Myocardial microvascular endothelial cells

## Abstract

**Background:**

This study explored the protective effects of melatonin on the hypertensive model in myocardial microvascular endothelial cells.

**Methods:**

Mouse myocardial microvascular endothelial cells were intervened with angiotensin II to establish hypertensive cell model and divided into control, hypertension (HP), hypertension + adenovirus negative control (HP + Ad-NC), hypertension + adenovirus carrying Mst1 (HP + Ad-Mst1), hypertension + melatonin (HP + MT), hypertension + adenovirus negative control + melatonin (HP + Ad-NC + MT), and hypertension + adenovirus carrying Mst1 + melatonin (HP + Ad-Mst1 + MT) groups. Autophagosomes were observed by transmission electron microscope. Mitochondrial membrane potential was detected by JC-1 staining. Apoptosis was detected by flow cytometry. Oxidative stress markers of MDA, SOD and GSH-PX were measured. The expression of LC3 and p62 was detected by immunofluorescence. Expression levels of Mst1, p-Mst1, Beclin1, LC3, and P62 were detected with Western blot.

**Results:**

Compared with the control group, the autophagosomes in HP, HP + Ad-Mst1, and HP + Ad-NC groups were significantly reduced. Compared with HP group, the autophagosomes in HP + Ad-Mst1 group were significantly reduced. The apoptosis of HP + MT group was significantly lower than HP group. Compared with HP + Ad-Mst1 group, the apoptosis of HP + Ad-Mst1 + MT group was significantly reduced. The ratio of JC-1 monomer in HP + MT group was significantly lower than HP group. Compared with HP + Ad-Mst1 group, the mitochondrial membrane potential of HP + Ad-Mst1 + MT group was also significantly reduced. MDA content in HP + MT group was significantly reduced, but SOD and GSH-PX activities were significantly increased. Compared with HP + Ad-Mst1 group, MDA content in HP + Ad-Mst1 + MT group was significantly reduced, whereas SOD and GSH-PX activities were increased significantly. Mst1 and p-Mst1 proteins in HP + MT group were significantly reduced. Compared with HP + Ad-Mst1 group, Mst1 and p-Mst1 in HP + Ad-Mst1 + MT group were reduced. P62 level was significantly decreased, while Beclin1 and LC3II levels were significantly increased. P62 in HP + MT group was significantly reduced, while Beclin1 and LC3II were significantly increased. Compared with HP + Ad-Mst1 group, P62 in HP + Ad-Mst1 + MT group was significantly reduced, but Beclin1 and LC3II were significantly increased.

**Conclusion:**

Melatonin may inhibit apoptosis, increase mitochondrial membrane potential, and increase autophagy of myocardial microvascular endothelial cells under hypertensive state via inhibiting Mst1 expression, thereby exerting myocardial protective effect.

**Supplementary Information:**

The online version contains supplementary material available at 10.1186/s12872-023-03159-1.

## Background

Hypertension is a critical risk factor for cardiovascular disease [1]. The prevalence of hypertension has been increasing worldwide year by year. In China, the hypertension prevalence among adults aged ≥ 18 years is 23.2%, with the case number of 245 million [2]. Hypertension can cause damages to many organs, including the heart, kidney, brain, eyes, etc. [3, 4]. Among them, hypertensive cardiomyopathy is the common pathological basis and independent risk factor for cardiovascular adverse events (such as heart failure and malignant arrhythmia). Recent studies have shown that the increase of microvascular endothelial cell apoptosis is also an important cause of hypertension [5, 6]. The apoptosis of microvascular endothelial cells leads to the scarcity of microvessels and the reduction of blood parallel pathways, which directly increases the peripheral resistance and increases the blood pressure. Therefore, how to prevent and delay myocardial injury in hypertensive patients is the core issue in hypertension management.

Melatonin is mainly secreted by the mammalian pineal gland. Its secretion is controlled by the sympathetic nervous system, showing a circadian rhythm. Melatonin is highly lipophilic, which can enter almost every cell in the body through the circulatory system [7, 8]. Melatonin exerts its biological effects through receptor- and non-receptor-mediated forms [9–11]. Studies have shown that melatonin has anti-inflammatory, anti-oxidative stress, anti-hypertension, anti-aging and anti-thrombotic effects, and can be used to prevent and treat cardiovascular diseases [12–19]. Evidence has accumulated for the protective actions of physiological concentrations of melatonin on microcirculation via maintaining the endothelial barrier function [20], preserving endothelial permeability [21] reducing cellular excessive oxidative stress [22], and alleviating endothelial-dependent NO overproduction [23]. In a mouse model of microvascular ischemia/reperfusion injury after percutaneous coronary intervention [24], melatonin has been shown to markedly reduce infarcted area, improve cardiac function, restore blood flow, and lower microcirculation perfusion defects. However, the biological role and mechanism of melatonin in hypertensive cardiomyopathy are still unclear.

As a key component in the Hippo pathway, mammalian STE20-like kinase 1 (Mst1) regulates apoptosis and cell proliferation. Mst1 is present in cardiac tissue, which determines cardiomyocyte fate by regulating autophagy. In a diabetic cardiomyopathy mouse model, Mst1 knockout restored autophagy and protected against apoptosis in cardiomyocytes, protecting against diabetic cardiomyopathy [25]. Recent studies have shown that Mst1 signaling regulates many cardiovascular diseases, including the aortic dissection, aortic aneurysm, atherosclerosis and myocardial infarction ischemic injury [26, 27]. Apoptosis and autophagy are important processes that regulate the occurrence and development of cardiovascular diseases. In addition to promoting apoptosis, Mst1 also inhibits autophagy. It has been reported that Mst1 can promote cardiac dysfunction in myocardial infarction mice by inhibiting autophagy. Mst1 plays a major role in the development of cardiac dysfunction by inhibiting autophagy and promoting the accumulation of protein aggregates [28]. In a diabetic cardiomyopathy mouse model [29], endogenous activation of melatonin may hamper the progression of diabetic cardiomyopathy by up-regulating cardiomyocyte autophagy through Mst1 inhibition.

In this study, melatonin was used to intervene the hypertensive model in myocardial microvascular endothelial cells. The action mechanisms of melatonin was also studied herein.

## Methods

### Cell lines and cell culture

Mouse myocardial microvascular endothelial cells were purchased from Procell Life Science & Technology (Wuhan, China). They were cultured with endothelial cell culture medium (CM-M129; Procell) containing 10% fetal bovine serum (FND500; Excell Bio), 100 U/ml penicillin (15070-063; Gibco) and 100 ug/ml streptomycin (Gibco).

### Cell model establishment and grouping

According to a previous study [10], the in vitro hypertensive cell model was established in mouse myocardial microvascular endothelial cells using angiotensin II (Ang II) (100 µM; AS-20,633; Anaspec Inc). According to different treatments, the cells were divided into the following seven groups: (1) the control group, in which the cells were cultured conventionally without any treatment for 48 h; (2) the HP group (hypertension group), in which the cells were cultured with conventional culture for 24 h, followed by the treatment with 100 µM Ang II for 24 h; (3) the HP + Ad-NC (hypertension + adenovirus negative control) group, in which the cells were treated with 100 µM Ang II for 24 h, and meanwhile infected with the negative control CON267 adenovirus (infection MOI = 200; Genechem Co., Ltd, Shanghai, China) for 48 h; (4) the HP + Ad-Mst1 (hypertension + adenovirus carrying Mst1) group, in which the cells were treated with 100 µM Ang II for 24 h, and simultaneously infected with adenovirus carrying the CDS region of the Stk4 gene (i.e., Mst1) (AD-Stk4; MOI = 200; Genechem) for 24 h; (5) the HP + MT (hypertension + melatonin) group, in which, after the routine culture for 24 h, cells were treated with 1 mM melatonin (M5250; Sigma) for 24 h at 1 h before Ang II intervention; (6) the HP + Ad-NC + MT (hypertension + adenovirus negative control + melatonin) group, which was treated with 1 mM melatonin for 24 h at 1 h before intervention with Ang II, and simultaneously infected with the negative control CON267 adenovirus (MOI = 200; infected for 48 h; Genechem); and (7) the HP + Ad-Mst1 + MT (hypertension + adenovirus carrying Mst1 + melatonin) group, which was treated 1 mM melatonin for 24 h at 1 h before Ang II intervention, and simultaneously infected with AD-Stk4 adenovirus (MOI = 200; infected for 48 h; Genechem).

### Autophagosome observation

Auophagosomes were observed with transmission electron microscope. Briefly, after intervention, the cells were collected and 1 mL 2.5% glutaraldehyde was added. After rinsing, the cells were dehydrated with acetone, treated with 1% osmic acid at room temperature for 2 h, and embedded in epoxy resin. The embedded samples were dried, ultrathin sectioned, and stained with uranyl acetate and lead citrate for 15 min and 5 min, respectively. The sections were observed under a transmission electron microscope. Totally, 10 fields were randomly selected from each group to conduct semi quantitative analysis on the number of autophagosomes.

### Flow cytometry analysis of apoptosis

Apoptosis was detected with flow cytometry. Briefly, cells were collected after intervention. After washing, cells were re-suspended with 500 µL 1×Binding Buffer. Then, 5 µL Annexin V-PE and 10 µL 7-AAD were added and incubated at 4 °C in dark for 10 min. The fluorescence was detected with flow cytometer (LSRFortessa; BD) within 30 min. A negative control (untreated cells) was used to adjust the voltage of FSC, SSC and fluorescence channels, and a single staining tube was used to adjust the compensation of fluorescence channel under this voltage condition.

### Mitochondrial membrane potential detection

After the intervention, the cells were collected. After washing, cells were incubated 500 µL 1×JC-1 staining solution (Beyotime, Haimen, Jiangsu, China) at 37 °C for 30 min. After washing again, 500 µL PBS was added to re-suspend the cells. The fluorescence intensity of JC-1 aggregates (red fluorescence) and monomer (green fluorescence) was detected on a flow cytometer within 30 min. The ratio of green to red intensity (i.e., the ratio of JC-monomer) was calculated.

### Detection of malondialdehyde (MDA), superoxide dismutase (SOD) and glutathione peroxidase (GSH-Px)

Intracellular levels of MDA, SOD and GSH-Px were detected. Briefly, after intervention, cells were collected. The contents of MDA, SOD, and GSH-P was detected with corresponding kits (Jiancheng Bioengineering institute, Nanjing, China).

### Western blot analysis

After intervention, the cells were collected and subjected to protein extraction. After electrophoresis and membrane transfer, incubation with primary antibodies against Mst1 (3682 S; CST), phospho-Mst1 (Thr183)/MST2 (Thr180) (49,332 S; CST), Beclin-1 (3738 S; CST), p62 antibody (23,214 S; Cell Signalling), LC3-I and LC3-II antibody (2775 S; Cell Signalling) was performed. Primary antibodies were obtained from Cell Signalling Technology (Danvers, MA, USA). After that, the membrane was incubated with corresponding HRP-conjugated secondary antibodies (goat against rabbit, ab205718; and goat against mouse, ab205719; Abcam). Color development was performed and detected with the ChemiScope mini chemiluminescence instrument. β-actin was used as internal control.

### Immunofluorescence

After intervention, the cells were collected and seeded onto coverslips. After culturing for 24 h, the coverslips were taken out and washed. After blocking with 1% BSA at room temperature for 30 min, the coverslips were incubated with anti-p62 antibody (232,145; Cell Signalling) and anti-LC3B antibody (2775 S; Cell Signalling) at 37 °C for 2 h. After washing, goat anti-rabbit IgG H&L secondary antibody (Alexa Fluor® 488) was used to incubate the cells at 37 °C in dark for 1 h. After washing, staining with DAPI was performed at room temperature in dark for 3 min. Finally, the cells were observed and photographed under laser confocal microscope.

### Statistical analysis

Experiments were performed in triplicates. Data were expressed as mean ± SEM. SPSS 19.0 software was used for statistical analysis. One-way ANOVA was used for group comparison. GraphPad Prism 5.0 software was used for graphing. *P* < 0.05 indicated significant difference.

## Results

### Melatonin promotes autophagy in myocardial microvascular endothelial cells

**Fig. 1:**
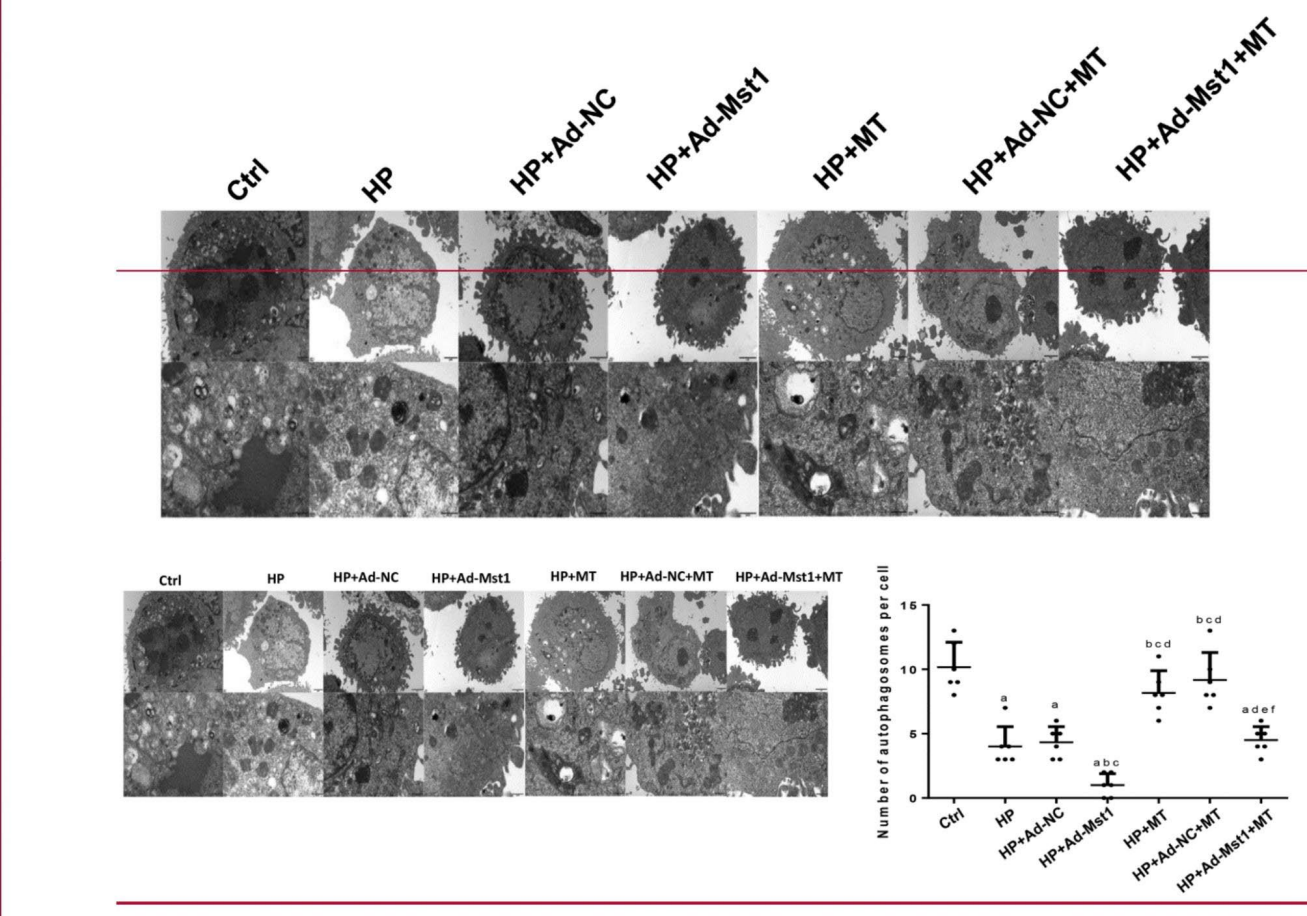
**Transmission electron microscopy analyzing autophagosomes in mouse myocardial microvascular endothelial cells.** Representative images of each group were shown on the left panel. Scale bar, 2 μm in upper panel, and 500 nm in lower panel. The number of autophagosomes per cell was shown on the right panel. Compared with control group, ^a^*P*<0.05; compared with HP group, ^b^*P*<0.05; compared with HP + Ad-NC group, ^c^*P*<0.05; compared with HP + Ad-Mst1 group, ^d^*P*<0.05; compared with HP + MT group, ^e^*P*<0.05; and compared with HP + Ad-NC + MT group, ^f^*P*<0.05

The numbers of autophagosomes in myocardial microvascular endothelial cells were observed by transmission electron microscope. As shown in Fig. 1, compared with the control group, the numbers of autophagosomes in the HP, HP + Ad-Mst1 and HP + Ad-NC groups were significantly reduced. Compared with the HP group, the number of autophagosomes in the HP + Ad-Mst1 group was significantly reduced. After the melatonin intervention, the number of autophagosomes was significantly increased, indicating that autophagy was enhanced. This data suggests that melatonin can promote autophagy.

### Melatonin alleviates apoptosis of myocardial microvascular endothelial cells caused by Mst1 over-expression

**Fig. 2:**
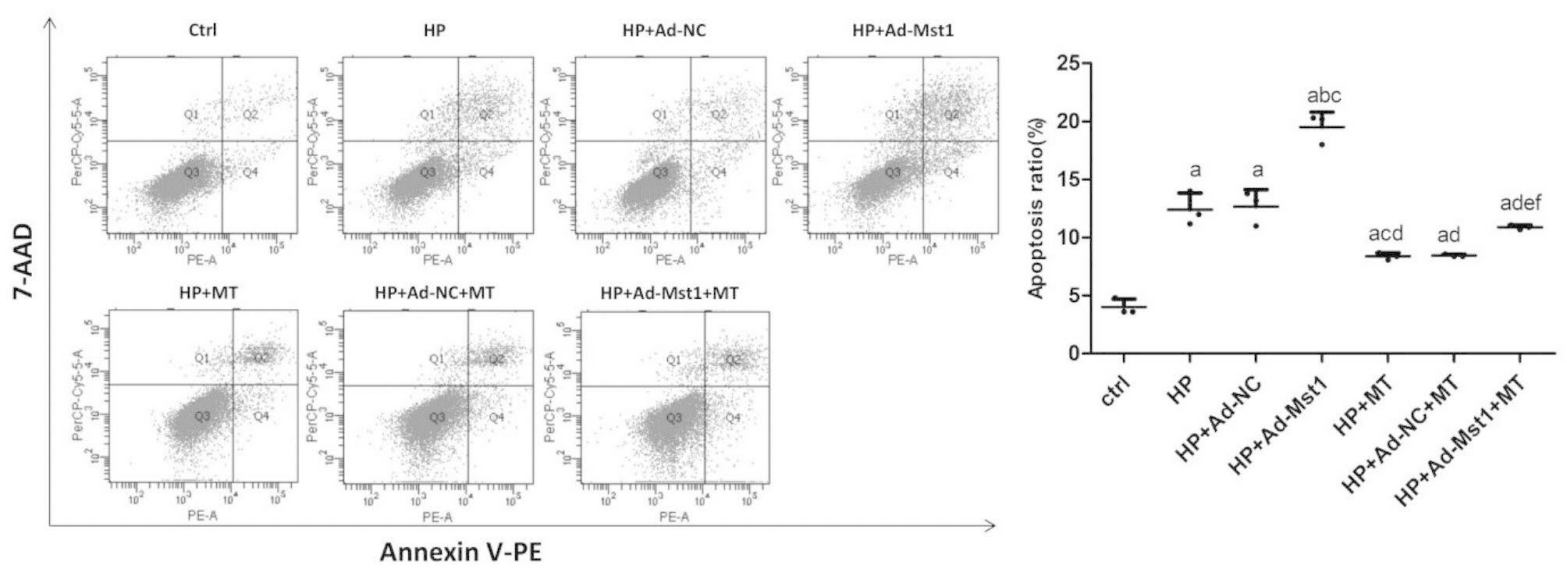
**Analysis of apoptosis rate of myocardial microvascular endothelial cells.** Flow cytometry was performed to analyze apoptosis of each group. Representative and quantitative results were shown. Early apoptotic cells (Annexin V-PE–positive and 7-AAD–negative cells) and late apoptotic cells (Annexin V-PE–positive and 7-AAD–positive cells) were gated. Q1, necrotic cells; Q2, late apoptotic cells; Q3, early stage apoptotic cells; and Q4, living cells. Compared with control group, ^a^*P*<0.05; compared with HP group, ^b^*P*<0.05; compared with HP + Ad-NC group, ^c^*P*<0.05; compared with HP + Ad-Mst1 group, ^d^*P*<0.05; compared with HP + MT group, ^e^*P*<0.05; and compared with HP + Ad-NC + MT group, ^f^*P*<0.05. n = 3

Apoptosis of the myocardial microvascular endothelial cells was detected by flow cytometry. As shown in Fig. 2, the apoptosis rate of the HP group was significantly increased than control group (*P* < 0.01). Compared with the HP group, after Mst1 over-expression, the apoptosis rate in HP + Ad-Mst-1 group was significantly increased (*P* < 0.01). After 1 mM melatonin was administered on the basis of Ang II intervention, the apoptosis rate of the HP + MT group was significantly lower than the HP group (*P* < 0.01). Compared with the HP + Ad-Mst1 group, the apoptosis of the HP + Ad-Mst1 + MT group was significantly decreased (*P* < 0.01). Thus, melatonin can alleviate the apoptosis of myocardial microvascular endothelial cells caused by Mst1 over-expression.

### Melatonin recovers decreased mitochondrial membrane potential induced by hypertension and Mst1 over-expression

**Fig. 3:**
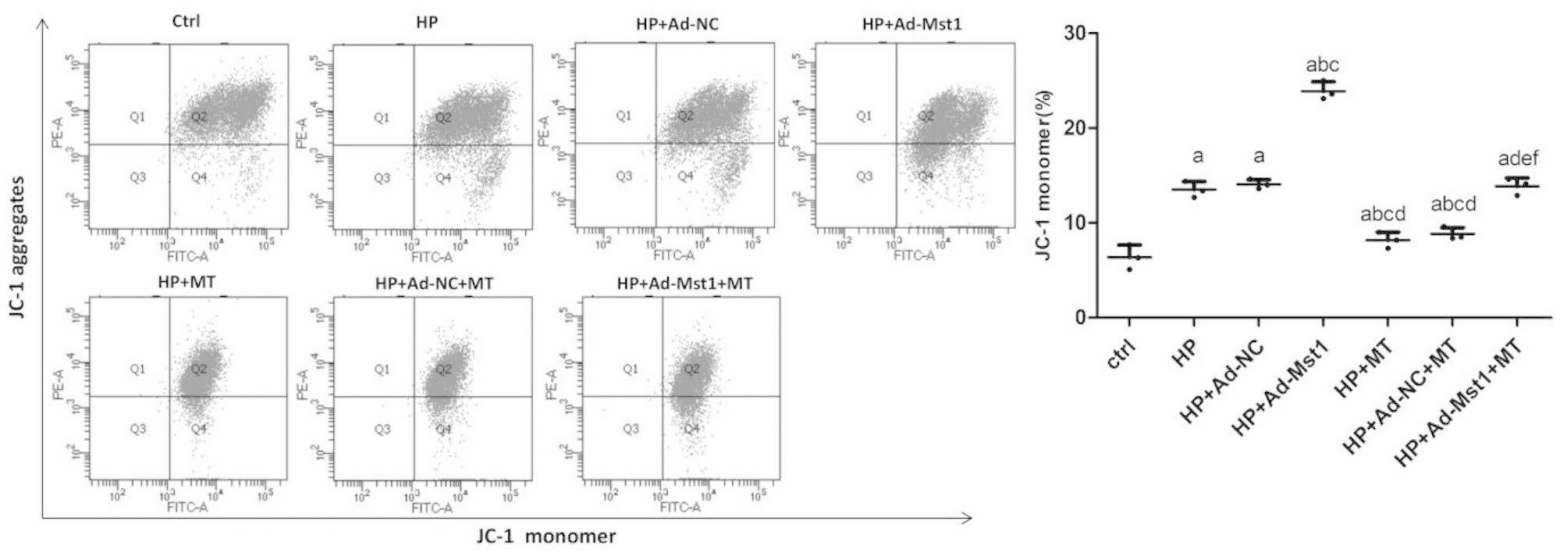
**Analysis of mitochondrial membrane potential in myocardial microvascular endothelial cells.** Flow cytometry was performed to analyze JC-staining of each group. Representative and quantitative results were shown. JC-monomer positive and JC-1 aggregate negative cells were gated (Q4). Compared with control group, ^a^*P*<0.05; compared with HP group, ^b^*P*<0.05; compared with HP + Ad-NC group, ^c^*P*<0.05; compared with HP + Ad-Mst1 group, ^d^*P*<0.05; compared with HP + MT group, ^e^*P*<0.05; and compared with HP + Ad-NC + MT group, ^f^*P*<0.05. n = 3

The mitochondrial membrane potential of myocardial microvascular endothelial cells was detected with the JC-1 staining. In cells with normal mitochondrial membrane potential, JC-1 spontaneously form aggregates that produce red fluorescence, whereas, in cells with reduced mitochondrial membrane potential, JC-1 cannot form aggregates and presents in monomer, which produces green fluorescence [30]. Thus, the elevated ratio of green to red fluorescence intensity, i.e. the elevated ratio of JC-1 monomer, represents the decrease of the mitochondrial membrane potential, and verse versa. As shown in Fig. 3, the ratio of JC-1 monomer in the HP group was significantly increased (*P* < 0.05), indicating significantly reduced mitochondrial membrane potential. Compared with the HP group, the ratio of JC-1 monomers was significantly increased after the Mst1 over-expression in HP + Ad-Mst-1 group (*P* < 0.05), indicating that the Mst1 over-expression could significantly reduce mitochondrial membrane potential. The ratio of JC-1 monomer in HP + MT group and HP + Ad-Mst1 + MT group was significantly lower than that in the HP group and HP + Ad-Mst1 group, respectively (*P* < 0.05), suggesting significantly increased mitochondrial membrane potential in HP + MT and HP + Ad-Mst1 + MT groups. Therefore, melatonin can recover the decreased mitochondrial membrane potential induced by hypertension and Mst1 over-expression.

### Melatonin inhibits increase of MDA and decrease of SOD and GSH-PX activity induced by hypertension and Mst1 over-expression

**Fig. 4:**
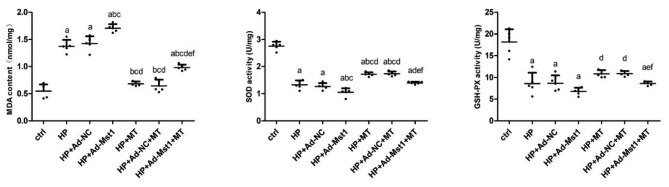
A**nalysis of MDA, SOD, and GSH-PX contents in myocardial microvascular endothelial cells.** Compared with control group, ^a^*P*<0.05; compared with HP group, ^b^*P*<0.05; compared with HP + Ad-NC group, ^c^*P*<0.05; compared with HP + Ad-Mst1 group, ^d^*P*<0.05; compared with HP + MT group, ^e^*P*<0.05; and compared with HP + Ad-NC + MT group, ^f^*P*<0.05. n = 3

The levels of MDA, SOD and GSH-PX in myocardial microvascular endothelial cells were detected. As shown in Fig. 4, compared with the control group, the MDA content in the HP group was significantly increased (*P* < 0.05), while the activities of SOD and GSH-PX were significantly decreased (*P* < 0.05). Compared with the HP group, the content of MDA in the HP + Ad-Mst1 group was significantly increased (*P* < 0.05), whereas the activities of SOD and GSH-PX were significantly decreased (*P* < 0.05). These results suggest that Mst1 over-expression significantly induces the production of MDA, but inhibits the activity of SOD and GSH-PX. After melatonin intervention on the basis of Ang II intervention, the HP + MT group had significantly decreased MDA (*P* < 0.05), but significantly increased activities of SOD and GSH-PX (*P* < 0.05). Compared with the HP + Ad-Mst1 group, there were significantly decreased MDA contents (*P* < 0.05), but significantly increased activities of SOD and GSH-PX (*P* < 0.05) in the HP + Ad-Mst1 + MT group. Hence, melatonin can significantly inhibit the increase of MDA and the decrease of SOD and GSH-PX activity induced by hypertension and Mst1 over-expression.

### Melatonin inhibits expression level of Mst1 and increases autophagy

**Fig. 5:**
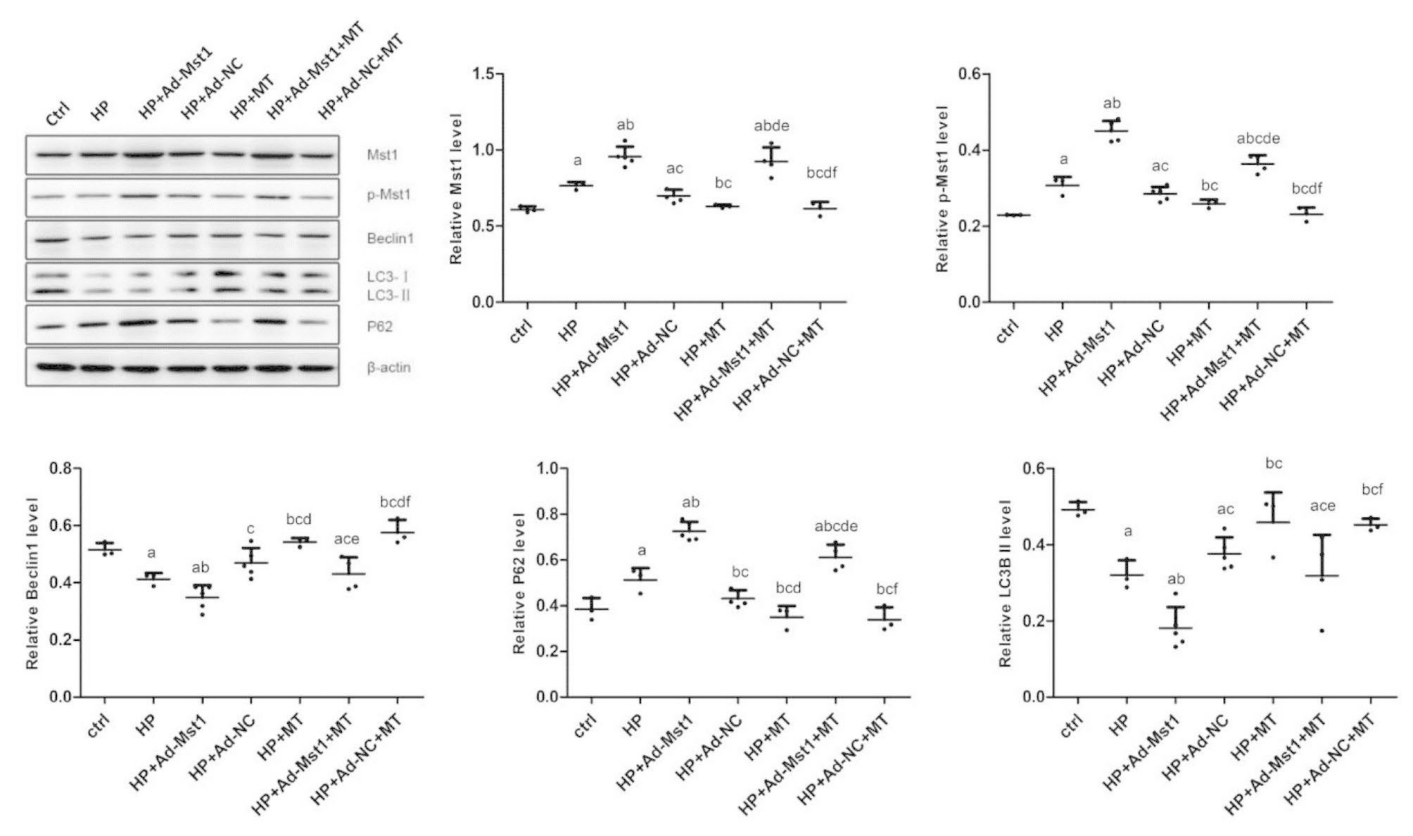
**Analysis of protein expression levels of Mst1, p-Mst1, Beclin1, P62, and LC3II in myocardial microvascular endothelial cells.** Protein expression levels of Mst1, p-Mst1, Beclin1, P62, and LC3II in myocardial microvascular endothelial cells were detected with Western blot analysis. Representative and quantitative results were shown. Uncropped protein blots are provided in the additional file1. Compared with control group, ^a^*P*<0.05; compared with HP group, ^b^*P*<0.05; compared with HP + Ad-NC group, ^c^*P*<0.05; compared with HP + Ad-Mst1 group, ^d^*P*<0.05; compared with HP + MT group, ^e^*P*<0.05; and compared with HP + Ad-NC + MT group, ^f^*P*<0.05. n = 3

The protein expression levels of the Mst1, p-Mst1, Beclin1, LC3, and P62 were analyzed by the Western blot analysis. As shown in Fig. 5, compared with the control group, the expression levels of Mst1 and p-Mst1 in the HP group were significantly increased (*P* < 0.05), suggesting that Ang II intervention could significantly up-regulate the expression of Mst1. Meanwhile, the expression levels of P62 in the HP group were significantly increased, and the protein levels of Beclin1 and LC3II were significantly decreased (*P* < 0.05), indicating that Ang II intervention significantly inhibits the level of autophagy. Compared with the HP group, the expression levels of Mst1, p-Mst1 and P62 in the HP + Ad-Mst1 group were significantly increased (*P* < 0.05), but the protein expression levels of Beclin1 and LC3II were significantly decreased (*P* < 0.05), indicating that the up-regulation of Mst1 could significantly inhibit the occurrence of autophagy. Moreover, after melatonin intervention on the basis of Ang II intervention, Mst1 and p-Mst1 protein expression levels in the HP + MT group were significantly decreased than HP group (*P* < 0.05). However, the autophagy-related protein P62 expression was significantly decreased, while the protein levels of Beclin1 and LC3II were significantly increased (*P* < 0.05). These results suggest that melatonin can inhibit the expression of Mst1 and promote the occurrence of autophagy. Compared with the HP + Ad-Mst1 group, the expression levels of Mst1 and p-Mst1 in the HP + Ad-Mst1 + MT group were decreased (*P* < 0.05). The expression level of P62 was significantly decreased, and the expression levels of Beclin1 and LC3II protein levels were significantly increased (*P* < 0.05). Thus, melatonin can inhibit the expression level of Mst1 and induce the increase of autophagy.

### Melatonin induces autophagy in myocardial microvascular endothelial cells

**Fig. 6:**
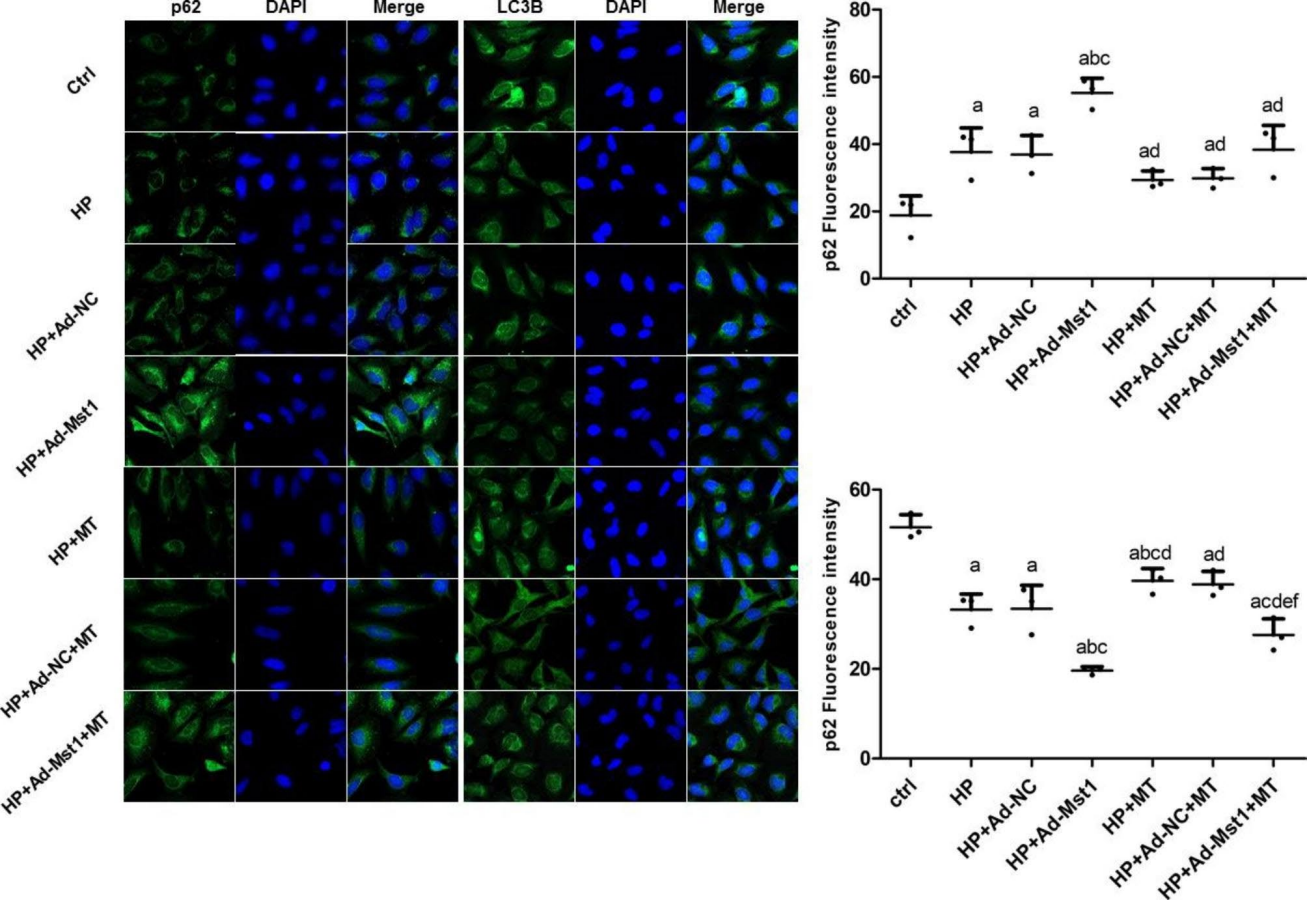
**Analysis of p62 and LC3B expression levels in mouse myocardial microvascular endothelial cells.** The expressions of p62 and LC3B in mouse myocardial microvascular endothelial cells were detected with immunofluorescence (200×). Representative and quantitative results were shown. Compared with control group, ^a^*P*<0.05; compared with HP group, ^b^*P*<0.05; compared with HP + Ad-NC group, ^c^*P*<0.05; compared with HP + Ad-Mst1 group, ^d^*P*<0.05; compared with HP + MT group, ^e^*P*<0.05; and compared with HP + Ad-NC + MT group, ^f^*P*<0.05. n = 3

Expression levels of LC3 and P62 in myocardial microvascular endothelial cells were further detected with immunofluorescence. As shown in Fig. 6, compared with the control group, the expression level of P62 in the HP group was significantly increased (*P* < 0.05). The protein expression levels of LC3B were significantly decreased (*P* < 0.05), indicating that Ang II intervention significantly inhibits the autophagy. HP + Ad-Mst1 group had significantly increased P62 but decreased LC3B protein expression levels than HP group (*P* < 0.05), indicating that the up-regulation of Mst1 can significantly inhibit the occurrence of autophagy. Meanwhile, after melatonin intervention on the basis of Ang II intervention, the autophagy-related protein P62 in the HP + MT group was significantly decreased, and LC3B was significantly increased than HP group (*P* < 0.05). Compared with the HP + Ad-Mst1 group, the HP + Ad-Mst1 + MT group had a significant decreased P62 expression, and a significant increase in the protein expression levels of LC3B (*P* < 0.05). Therefore, melatonin can induce the increase of autophagy.

## Discussion

Studies have reported that melatonin can alleviate myocardial ischemia/reperfusion injury [31, 32], inhibit the production of ROS, improve cardiac contractility, and reduce cardiomyocyte apoptosis [15, 33, 34]. However, there is no report on the mechanism of melatonin in myocardial injury in hypertensive patients. Mst1, which is present in cardiac tissue and determines cardiomyocyte fate by regulating autophagy, plays a critical role in cardiac and vascular homeostasis in the cardiovascular system [35, 36] Autophagy is particularly important in maintaining cardiomyocyte homeostasis. However, defective or excessive autophagy could lead to cardiovascular diseases, including the heart failure and atherosclerosis [37, 38]. In a study using the mouse model of diabetic cardiomyopathy [25], the results showed that melatonin inhibited the expression of p-Mst1 and Mst1, and up-regulated the autophagy in myocardial tissue of diabetic mice, suggesting that melatonin may exert its cardio protective effect by inhibiting Mst1. However, whether melatonin is involved in hypertensive myocardial microvascular endothelial cell damage remains unclear. In this study, Ang II (100 µM) was used to intervene mouse myocardial microvascular endothelial cells for 24 h to establish the hypertensive cell model. The concentration of 1 mM was used for the melatonin intervention. We found that Mst1 over-expression increased apoptosis and weakened autophagy of the hypertensive cell model, while melatonin significantly inhibited apoptosis and promoted autophagy. These results suggest that melatonin may inhibit Mst1 expression, induce autophagy, and inhibit apoptosis of myocardial microvascular endothelial cells under hypertensive state, thereby further reducing injury to myocardial microvascular endothelial cells.

Mitochondria constitute approximately 45% of the myocardial volume [39, 40]. More than 90% of ATP generated by mitochondrial respiration is utilized by cardiomyocytes [41]. It has been hypothesized that mitochondria are the original sites of melatonin biosynthesis [42]. The decrease in mitochondrial membrane potential is a hallmark event in the early stages of apoptosis. JC-1 is an ideal fluorescent probe widely used to detect mitochondrial membrane potential. Herein, we found that the proportion of JC-1 monomers in the HP + MT group was significantly lower than the HP group. Compared with the HP + Ad-Mst1 group, the mitochondrial membrane potential of the HP + Ad-Mst1 + MT group was also significantly decreased. These results suggest that melatonin can alleviate the decreased mitochondrial membrane potential induced by hypertension and Mst1 over-expression. The P62 level is negatively correlated with the autophagy level, and decreased autophagy could cause the accumulation of P62 [43]. LC3 is a key protein in autophagy, representing the autophagy strength [43]. The expression level of P62 is often combined with LC3 as an indicator to evaluate the level of autophagy [43]. Beclin1 is a Bcl-2 homology 3 domain protein that plays an important role in the initiation of autophagy [41, 44]. In a mouse model of diabetic cardiomyopathy [43], melatonin has been shown to inhibit Mst1 expression and Mst1 phosphorylation, up-regulate LC3-II expression, and reduce p62 accumulation, suggesting that melatonin may up-regulate cardiomyocyte autophagy by inhibiting Mst1, thereby inhibiting the progression of diabetic cardiomyopathy. An increasing number of studies [45, 46] have confirmed the anti-apoptotic effect of melatonin in preventing cardiomyocyte injury. In this study, we detected the protein expressions of Mst1, p-Mst1, Beclin1, LC3 and P62 in mouse myocardial microvascular endothelial cells. The results showed that after melatonin intervention on the basis of Ang II intervention, Mst1 and p-Mst1 protein expression levels were significantly reduced. However, the expression levels of P62 were significantly decreased, and those of Beclin1 and LC3II were significantly increased. Compared with the HP + Ad-Mst1 group, the expressions of Mst1 and p-Mst1 in the HP + Ad-Mst1 + MT group were decreased. Similarly, P62 was significantly decreased, and Beclin1 and LC3II protein levels were significantly increased. These results confirm that melatonin can inhibit the expression of Mst1 and induce the increase of autophagy.

Oxidative stress mainly refers to the imbalance of oxidation and anti-oxidation under stimuli and plays an important role in hypertension [47]. MDA is one of the products of lipid peroxidation and is one of the biomarkers of oxidative stress [48]. SOD can inhibit damage to cells by •O2-, thus protecting cellular structural integrity [49]. In this study, compared with the HP group, the MDA content in the HP + Ad-Mst1 group was up-regulated significantly, while the SOD and GSH-PX activities were down-regulated significantly. Therefore, Mst1 over-expression significantly induces the production of MDA, and inhibits the activity of SOD and GSH-PX, thus aggravating cell damages. After melatonin intervention on the basis of Ang II treatment, the content of MDA in HP + MT group decreased significantly, and the activities of SOD and GSH-PX increased significantly. Compared with the HP + Ad-Mst1 group, the MDA content in the HP + Ad-Mst1 + MT group reduced significantly, and the SOD and GSH-PX activities elevated significantly. These results suggest that melatonin can inhibit the increase of MDA and reverse the decrease of SOD and GSH-PX activity induced by Ang II and Mst1 over-expression.

## Conclusion

**Fig. 7:**
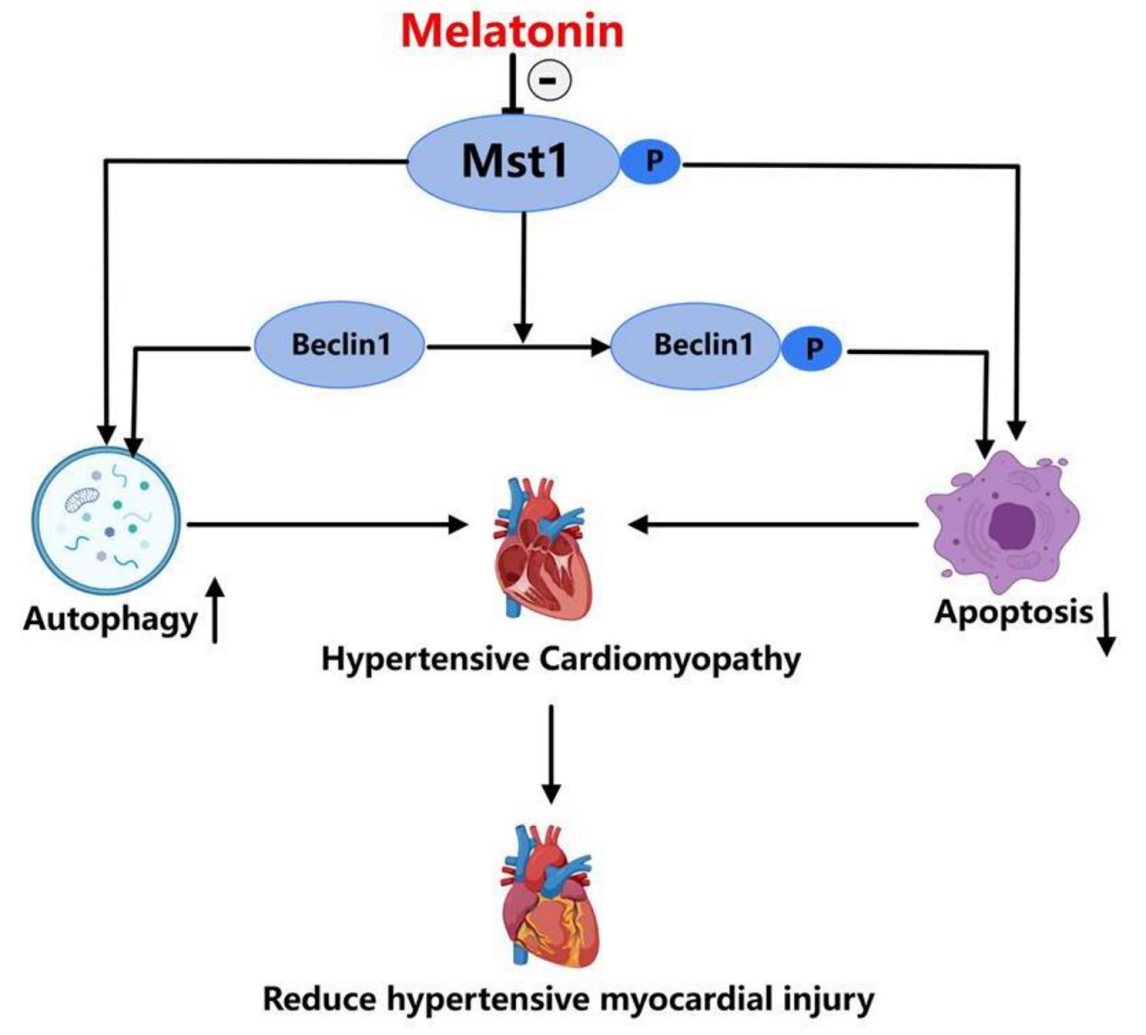
**The schematic figure showing the mechanism underlying the protective role of melatonin in hypertensive cell model.** Melatonin could inhibit Mst1 expression in myocardial microvascular endothelial cells under hypertensive state, which then may inhibit apoptosis, and increase autophagy of myocardial microvascular endothelial cells, thereby exerting myocardial protective effect

In conclusion, our results showed that melatonin inhibited Mst1 expression, promoted autophagy, and reduced the apoptosis rate of myocardial microvascular endothelial cells (Fig. 7). Meanwhile, melatonin increased the mitochondrial membrane potential and promoted the generation of autophagosomes in myocardial microvascular endothelial cells. Therefore, our results may provide new insights into the role of melatonin in autophagy and apoptosis in myocardial microvascular endothelial cells, and may provide new ideas and targets for the diagnosis and treatment of hypertensive cardiomyopathy. However, the specific mechanisms underlying these effects of melatonin are unclear and should be further studied.

## Electronic supplementary material

Below is the link to the electronic supplementary material.

Additional File: Uncropped protein blots

## Data Availability

The datasets generated and/or analysed during the current study are not publicly available but are available from the corresponding author on reasonable request.

## List of Abbreviations

Mst1: mammalian STE20-like kinase 1
MDA: malondialdehyde
SOD: superoxide dismutase
GSH-P: glutathione peroxidase
HP group: hypertension group
HP + Ad-NC group: hypertension + adenovirus negative control group
HP + Ad-Mst1 group: hypertension + adenovirus carrying Mst1 group
HP + MT group: hypertension + melatonin group
HP + Ad-NC + MT group: hypertension + adenovirus negative control + melatonin group
HP + Ad-Mst1 + MT group: hypertension + adenovirus carrying Mst1 + melatonin group
