# Supplementary material for: Protective effects of melatonin on myocardial microvascular endothelial cell injury under hypertensive state by regulating Mst1
Source: BMC Cardiovasc Disord. 2023 Apr 1;23:179. doi: 10.1186/s12872-023-03159-1 (PMC10068162; doi:10.1186/s12872-023-03159-1)
Supplement: Supplementary file 1 — Additional File: Uncropped protein blots [file 12872_2023_3159_MOESM1_ESM.ppt]

## Slide 1
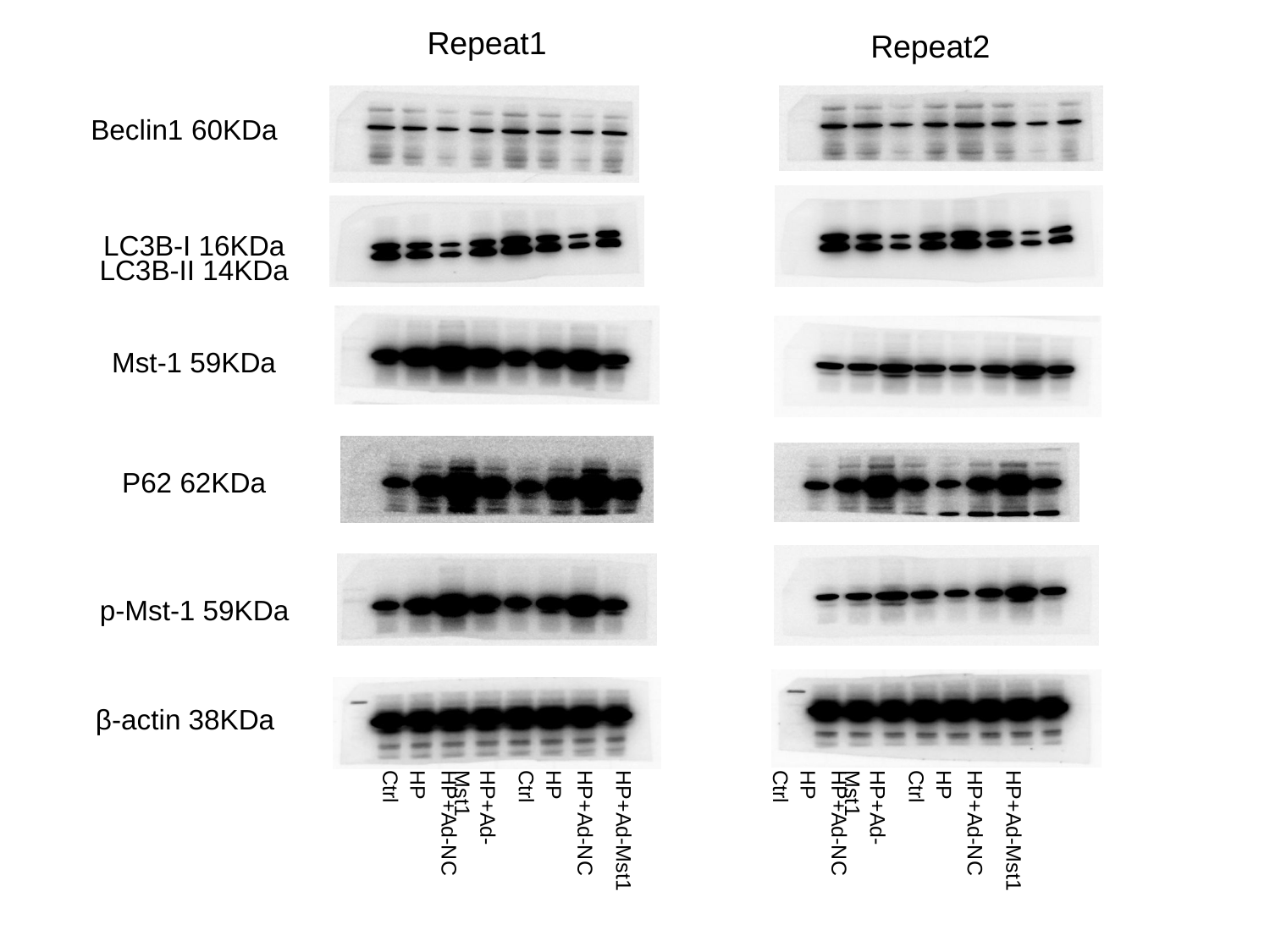

Repeat1
Repeat2
Beclin1 60KDa
LC3B-I 16KDa
LC3B-II 14KDa
Mst-1 59KDa
P62 62KDa
p-Mst-1 59KDa
β-actin 38KDa
Ctrl
HP
HP+Ad-NC
HP+Ad-Mst1
Ctrl
HP
HP+Ad-NC
HP+Ad-Mst1
Ctrl
HP
HP+Ad-NC
HP+Ad-Mst1
Ctrl
HP
HP+Ad-NC
HP+Ad-Mst1

## Slide 2
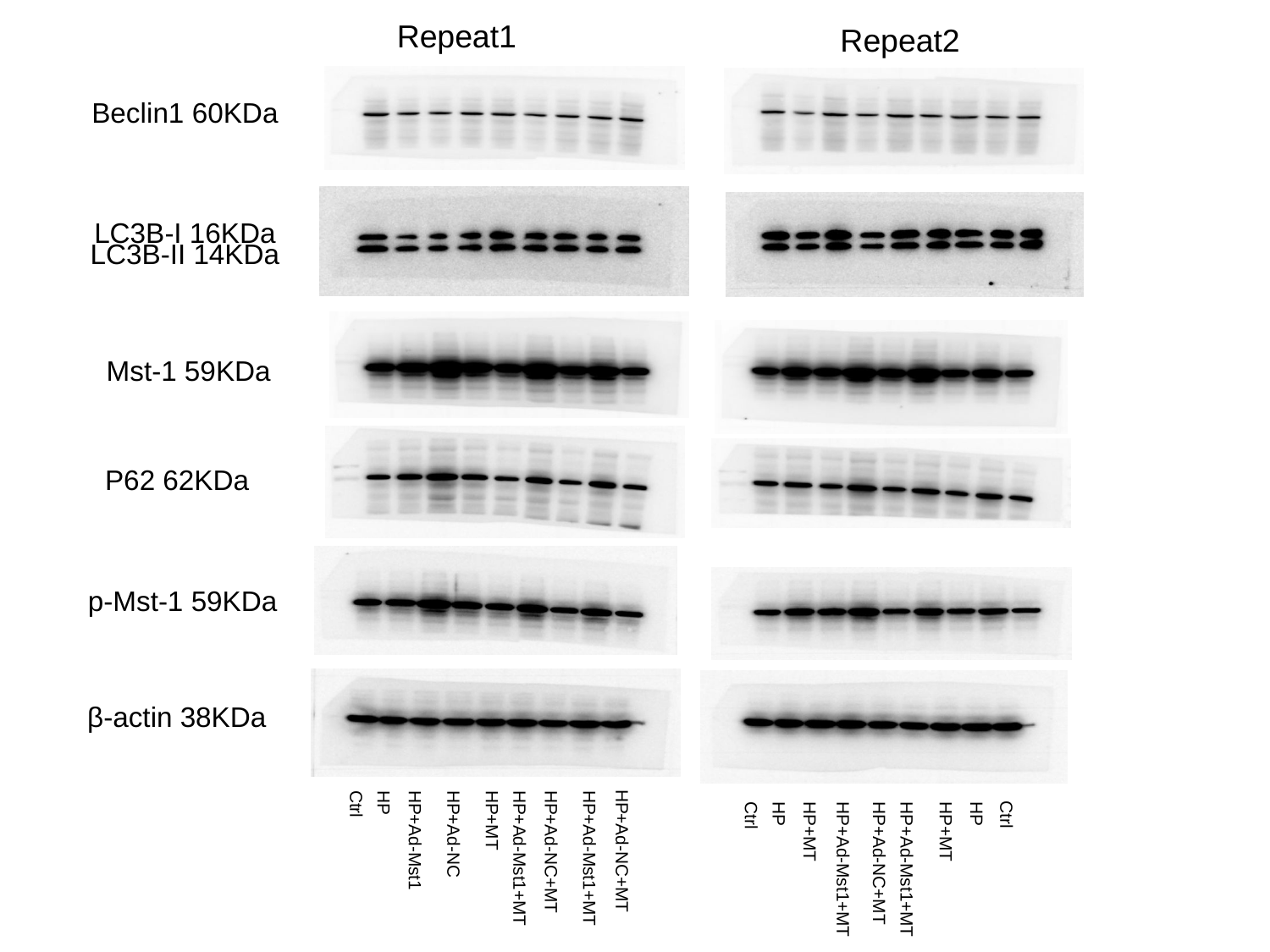

Repeat1
Repeat2
Beclin1 60KDa
LC3B-I 16KDa
LC3B-II 14KDa
Mst-1 59KDa
P62 62KDa
p-Mst-1 59KDa
β-actin 38KDa
HP+Ad-NC+MT
Ctrl
HP
HP+Ad-Mst1
HP+Ad-NC
HP+MT
HP+Ad-Mst1+MT
HP+Ad-NC+MT
HP+Ad-Mst1+MT
Ctrl
Ctrl
HP
HP+MT
HP+Ad-Mst1+MT
HP+Ad-NC+MT
HP+Ad-Mst1+MT
HP+MT
HP
